# Supplementary material for: Effective population size for culturally evolving traits
Source: PLoS Comput Biol. 2022 Apr 8;18(4):e1009430. doi: 10.1371/journal.pcbi.1009430 (PMC9020689; doi:10.1371/journal.pcbi.1009430)
Supplement: S1 Text — (PDF) [file pcbi.1009430.s001.pdf]

S1 Text for

"Effective population size for culturally evolving traits"

Dominik Deffner[1,2,3*], Anne Kandler[1] & Laurel Fogarty[1]

[1]Department of Human Behavior, Ecology and Culture, Max Planck Institute for Evolutionary Anthropology, Leipzig, Germany
[2]Science of Intelligence Excellence Cluster, Technical University Berlin, Berlin, Germany
[3]Center for Adaptive Rationality, Max Planck Institute for Human Development, Berlin, Germany
*Corresponding author: deffner@mpib-berlin.mpg.de

Calculations for example used in section "What is effective population size and why do we need it?"

Remember that we assumed there are two populations, A and B, with current census population sizes of 1000 and 500 individuals, respectively. Population A had a population bottleneck ten generations ago when its census size fell to just 10 individuals before immediately returning to its current size of 1000. If population size is fluctuating over time and we have information from a total of T non-overlapping generations, the effective size N_e is given by the harmonic mean (i.e. the reciprocal of the arithmetic mean of the reciprocals) of the population sizes at each point in time t:

$$N_e = \frac{1}{\frac{1}{T}\sum_{t=1}^{T}\frac{1}{N_t}}. \tag{A1}$$

In our example, for population A, $N_1 = 10$ and $N_{[2:10]} = 1000$, for population B, $N_{[1:10]} = 500$. Plugging these values into Eq. (A1) gives the effective population sizes of $N_e = 91.7$ for population A and $N_e = 500$ for population B.

Based on these effective sizes and assuming a certain innovation rate (here $\mu = 0.1$) and unbiased cultural transmission, we can use results from population genetics (see [1], p.115) to approximate the mean number of cultural traits we expect to see each generation, $E(K)$, as follows:

$$E(K) \approx \theta + \int_{N_e^{-1}}^{1} \theta x^{-1}(1-x)^{\theta-1}dx, \tag{A2}$$

where $\theta = 2N_e\mu$ is the standard population mutation parameter. For population B with $N_e = 500$, the expected number of traits is, thus, $E(K) \approx 223$. For population A with $N_e = 91.7$, we expect to see on average $E(K) \approx 41$ traits in a given generation.

REFERENCES

1. Ewens WJ. Mathematical population genetics 1: theoretical introduction. volume 27. Springer Science Business Media, 2012.
