# Supplementary material for: Effective population size for culturally evolving traits
Source: PLoS Comput Biol. 2022 Apr 8;18(4):e1009430. doi: 10.1371/journal.pcbi.1009430 (PMC9020689; doi:10.1371/journal.pcbi.1009430)
Supplement: S2 Text — (PDF) [file pcbi.1009430.s002.pdf]

S2 Text for

"Effective population size for culturally evolving traits"

Dominik Deffner[1,2,3*], Anne Kandler[1] & Laurel Fogarty[1]

[1]Department of Human Behavior, Ecology and Culture, Max Planck Institute for Evolutionary Anthropology, Leipzig, Germany

[2]Science of Intelligence Excellence Cluster, Technical University Berlin, Berlin, Germany

[3]Center for Adaptive Rationality, Max Planck Institute for Human Development, Berlin, Germany

[*]Corresponding author: deffner@mpib-berlin.mpg.de

Properties of the haploid Wright-Fisher population

Both mean and variance in offspring number are roughly 1 for a haploid Wright-Fisher population. Why is this the case? For the Wright-Fisher population with a population size of N, N parents are chosen at random with replacement from the full population. From the point of view of any focal individual, the probability of being chosen as a parent to any one of the N offspring is $p = 1/N$, the probability of not being chosen, correspondingly, is $q = 1 - 1/N$. We conduct N trials and so the number of offspring produced by any member of the population is a binomially distributed random variable. The mean of this is simply $\bar{k} = Np = N/N = 1$. The variance is $\sigma^2 = Npq$. This is $\sigma^2 = N\left(\frac{1}{N}\right)\left(1 - \frac{1}{N}\right)$. Simplifying, we get $\sigma^2 = 1 - \frac{1}{N}$. It is common here to neglect terms on the order of $\frac{1}{N}$; they are very small because N is usually very large, giving us $\sigma^2 \approx 1$.
