# Supplementary material for: Effective population size for culturally evolving traits
Source: PLoS Comput Biol. 2022 Apr 8;18(4):e1009430. doi: 10.1371/journal.pcbi.1009430 (PMC9020689; doi:10.1371/journal.pcbi.1009430)
Supplement: S3 Text — (PDF) [file pcbi.1009430.s003.pdf]

S3 Text for

"Effective population size for culturally evolving traits"

Dominik Deffner[1,2,3*], Anne Kandler[1] & Laurel Fogarty[1]

[1]Department of Human Behavior, Ecology and Culture, Max Planck Institute for Evolutionary Anthropology, Leipzig, Germany
[2]Science of Intelligence Excellence Cluster, Technical University Berlin, Berlin, Germany
[3]Center for Adaptive Rationality, Max Planck Institute for Human Development, Berlin, Germany
[*]Corresponding author: deffner@mpib-berlin.mpg.de

Derivation of inbreeding and variance N_e

Different aspects of the evolution of the Wright-Fisher population have been used to define N_e. We seek expressions for both inbreeding and variance effective numbers for situations where (1) there is variation in offspring numbers and (2) population sizes might differ between parental and offspring generation. Here, we follow the general presentation of Kimura and Crow [1], but make adjustments for haploid populations where necessary. Haploids are characterized by only one set of variants, whereas diploids are characterized by two sets. In cultural transmission, learners adopt a single variant of a cultural trait from one or several role models, there is no inheritance of two corresponding alleles from sexually reproducing parents.

Consider a population of N_{t-1} individuals each contributing a variable number, k_i, of offspring to the next generation. In general, the mean number of offspring is

(C1) $$\bar{k} = \frac{\sum_{i=1}^{N_{t-1}} k_i}{N_{t-1}},$$

and the variance in offspring number is

(C2) $$\sigma^2 = \frac{\sum_{i=1}^{N_{t-1}} k_i^2}{N_{t-1}} - \bar{k}^2.$$

Inbreeding effective population size. The identity-by-descent (or inbreeding) effective population size N_e^i utilizes the fact that in finite populations there is a certain probability that two randomly selected individuals in generation t are descendant from the same parent. As this single-generation probability of identity by descent, P_t, in the ideal Wright-Fisher population is simply $1/N_{t-1}$, we can use an estimate of this probability in the real population to calculate the effective population size as $N_e^i = 1/P_t$. For our example, the number of ways in which two offspring from a given parent i can be selected is $k_i(k_i-1)/2$. Summing over all members of the parental generation, the total number of ways in which two offspring from the same parent can be selected is $\sum_{i=1}^{N_{t-1}} k_i(k_i-1)/2$, whereas the total number of offspring

pairs is $N_{t-1}\bar{k}(N_{t-1}\bar{k}-1)/2$. Dividing the former by the latter, P_t can thus be calculated as

$$P_t = \frac{\sum_{i=1}^{N_{t-1}} k_i(k_i-1)}{N_{t-1}\bar{k}(N_{t-1}\bar{k}-1)} = \frac{\sum_{i=1}^{N_{t-1}} k_i^2 - \sum_{i=1}^{N_{t-1}} k_i}{N_{t-1}\bar{k}(N_{t-1}\bar{k}-1)}. \tag{C3}$$

From the definition of the mean ($\bar{k}$; see equation C1),

$$\sum_{i=1}^{N_{t-1}} k_i = N_{t-1}\bar{k}. \tag{C4}$$

and from the definition of the variance (σ^2; see equation C2),

$$\sum_{i=1}^{N_{t-1}} k_i^2 = N_{t-1}\sigma^2 + N_{t-1}\bar{k}^2. \tag{C5}$$

Substituting these into equation C3 results in

$$P_t = \frac{N_{t-1}\sigma^2 + N_{t-1}\bar{k}^2 - N_{t-1}\bar{k}}{N_{t-1}\bar{k}(N_{t-1}\bar{k}-1)}. \tag{C6}$$

Rearranging, we can calculate the inbreeding effective number for haploid populations as

$$N_e^i = \frac{1}{P_t} = \frac{N_{t-1}\bar{k}-1}{\bar{k}-1+\frac{\sigma^2}{\bar{k}}}. \tag{C7}$$

Variance effective population size. The variance effective population size N_e^v, in contrast, focuses on the amount of random variation in allele frequencies from one generation to the next. Assume that p is the frequency of an allele in an ideal Wright-Fisher population of size N. The sampling variance of the gene frequency drift from parent to offspring generation is $V_{\delta p} = p(1-p)/N$ and, therefore, $N_e^v = p(1-p)/V_{\delta p}$. Similarly to above, we seek an expression for $V_{\delta p}$ to infer the corresponding effective number. Again, assume there is a population of N_{t-1} individuals each contributing a number of k_i offspring to the next generation. Let p denote the frequency of allele A in the population and let n_1 be the number of individuals carrying allele A ($n_1 = N_{t-1}p$). The number of A alleles contributed to the next generation is $\sum_{i=1}^{n_1} k_i$. Thus, the increment in A alleles from generation $t-1$ to generation t is given by:

$$N_{t-1}\bar{k}\delta p = \sum_{i=1}^{n_1} k_i - N_{t-1}\bar{k}p. \tag{C8}$$

Expressing p as $\sum_{1}^{n_1} \frac{\bar{k}}{N_{t-1}\bar{k}}$ and substituting into equation (C8) gives,

$$N_{t-1}\bar{k}\delta p = \sum_{i=1}^{n_1}(k_i - \bar{k}). \tag{C9}$$

Using expectation notation, we now write an expression for the variance in the change in p, $V_{\delta p} = \mathbf{E}[\delta p]^2$.

$$(N_{t-1}\bar{k})^2 V_{\delta p} = \mathbf{E}[\sum_{i=1}^{n_1}(k_i - \bar{k})]^2 \tag{C10}$$
$$= n_1\sigma^2 + n_1(n_1 - 1)C_{kk'},$$

where $C_{kk'} = -\frac{\sigma^2}{N_{t-1}-1}$ is the covariance in offspring number for randomly selected pairs from the parental generation. Substituting this and simplifying results in

$$= \frac{\sigma^2}{N_{t-1} - 1} n_1(N_{t-1} - n_1). \tag{C11}$$

If we then replace n_1 by $N_{t-1}p$, we get

$$(N_{t-1}\bar{k})^2 V_{\delta p} = \frac{\sigma^2}{N_{t-1} - 1} N_{t-1}p(N_{t-1} - N_{t-1}p), \tag{C12}$$

and

$$\bar{k}^2 V_{\delta p} = \frac{\sigma^2}{N_{t-1} - 1}(1 - p). \tag{C13}$$

Replacing $V_{\delta p}$ with $\frac{p(1-p)}{N_e^v}$ gives

$$\bar{k}^2 \frac{p(1 - p)}{N_e^v} = \frac{\sigma^2}{N_{t-1} - 1}(1 - p). \tag{C14}$$

Solving for N_e^v results in

$$N_e^v = \frac{(N_{t-1} - 1)\bar{k}^2}{\sigma^2} = \frac{(N_{t-1} - 1)\bar{k}}{\frac{\sigma^2}{\bar{k}}}. \tag{C15}$$

REFERENCES

1. Kimura M, Crow JF. The measurement of effective population number. Evolution, pages 279–288, 1963
