# Supplementary material for: Effective population size for culturally evolving traits
Source: PLoS Comput Biol. 2022 Apr 8;18(4):e1009430. doi: 10.1371/journal.pcbi.1009430 (PMC9020689; doi:10.1371/journal.pcbi.1009430)
Supplement: S4 Text — (PDF) [file pcbi.1009430.s004.pdf]

S4 Text for

"Effective population size for culturally evolving traits"

Dominik Deffner[1,2,3*], Anne Kandler[1] & Laurel Fogarty[1]

[1]Department of Human Behavior, Ecology and Culture, Max Planck Institute for Evolutionary Anthropology, Leipzig, Germany

[2]Science of Intelligence Excellence Cluster, Technical University Berlin, Berlin, Germany

[3]Center for Adaptive Rationality, Max Planck Institute for Human Development, Berlin, Germany

[*]Corresponding author: deffner@mpib-berlin.mpg.de

Burn-in details for simulation models

To remove any potential effect of initial conditions, before we start recording values, we first let the system evolve through unbiased transmission until it reaches its equilibrium state. To achieve this, we set up two separate populations, population A with maximum diversity (i.e., each individual carrying a different variant) and population B with 0 diversity (i.e., each individual carrying the same variant). We then recorded the Simpson Diversity Index ($D = 1 - \sum_{i=1}^{S}(n_i/N)^2$; see section on simulation set-up in the main text) in each generation and let the system evolve until diversity in population B first exceeds diversity in population A. This procedure ensures that the system has reached its equilibrium diversity value which is also confirmed by the observation that all simulation results reported in the main text were identical for both original populations.
