# Supplementary material for: Effective population size for culturally evolving traits
Source: PLoS Comput Biol. 2022 Apr 8;18(4):e1009430. doi: 10.1371/journal.pcbi.1009430 (PMC9020689; doi:10.1371/journal.pcbi.1009430)
Supplement: S5 Text — (PDF) [file pcbi.1009430.s005.pdf]

S5 Text for

"Effective population size for culturally evolving traits"

Dominik Deffner[1,2,3*], Anne Kandler[1] & Laurel Fogarty[1]

[1]Department of Human Behavior, Ecology and Culture, Max Planck Institute for Evolutionary Anthropology, Leipzig, Germany
[2]Science of Intelligence Excellence Cluster, Technical University Berlin, Berlin, Germany
[3]Center for Adaptive Rationality, Max Planck Institute for Human Development, Berlin, Germany
[*]Corresponding author: deffner@mpib-berlin.mpg.de

Expressing the variance of the full population in terms of sub-population variances and means

The combined variance, σ_c^2, is given by

$$\sigma_c^2 = \frac{1}{N}\sum_{i=1}^{N}(k_i - \bar{k}_c)^2, \tag{D1}$$

where, again, N is the total population size, k_i is the number of cultural offspring of the ith individual in the parental generation, and $\bar{k}_c$ is the combined mean offspring number for the whole population (including both the group of cultural transmitters and the group of non-transmitters). To write a general expression for the variance in the full population, we split the population into two arbitrary groups of size n_1 and n_2 where $n_1 + n_2 = N$. We label the mean offspring number within those subpopulations as $\bar{k}_1$ and $\bar{k}_2$ respectively, and the variances in offspring number, similarly, as σ_1^2 and σ_2^2.

Now, it is possible to express the variance of the complete population (σ_c^2) in terms of the means and variances of the two subpopulations.

$$\begin{aligned} N\sigma_c^2 &= \sum_{i=1}^{N}\left(k_i - \bar{k}_c\right)^2 \\ &= \sum_{i=1}^{n_1}\left(k_i - \bar{k}_c\right)^2 + \sum_{i=n_1+1}^{N}\left(k_i - \bar{k}_c\right)^2 . \end{aligned} \tag{D2}$$

With some rearranging, we get

$$\begin{aligned} N\sigma_c^2 &= \sum_{i=1}^{n_1}\left(\left(k_i - \bar{k}_1\right) - \left(\bar{k}_c - \bar{k}_1\right)\right)^2 + \sum_{i=n_1+1}^{N}\left(\left(k_i - \bar{k}_2\right) - \left(\bar{k}_c - \bar{k}_2\right)\right)^2 \\ &= \sum_{i=1}^{n_1}\left(k_i - \bar{k}_1\right)^2 + n_1\left(\bar{k}_c - \bar{k}_1\right)^2 + \sum_{i=n_1+1}^{N}\left(k_i - \bar{k}_2\right)^2 + n_2\left(\bar{k}_c - \bar{k}_2\right)^2 , \end{aligned} \tag{D3}$$

using the fact that in general $\sum_{j=1}^{M} k_j - \bar{k}_M = 0$. So, we have

$$N\sigma_c^2 = n_1\sigma_1^2 + n_2\sigma_2^2 + D_N, \tag{D4}$$

where

(D5) $$D_N = n_1\left(\bar{k}_1 - \bar{k}_c\right)^2 + n_2\left(\bar{k}_2 - \bar{k}_c\right)^2.$$

To allow some simplifications we can rewrite D_N as

(D6) $$D_N = \frac{n_1 n_2}{N}\left(\bar{k}_1 - \bar{k}_2\right)^2.$$

Details of this simplification can be found in O'Neill [1]. The decomposed expression for σ_c^2, then is

(D7) $$\sigma_c^2 = \frac{n_1\sigma_1^2 + n_2\sigma_2^2 + \frac{n_1 n_2}{N}\left(\bar{k}_1 - \bar{k}_2\right)^2}{N}.$$

Now, to apply this to our population and to the calculation of effective population size, we make the following assumptions. Population 1 is the transmitting population. Therefore, $n_1 = R, n_2 = (N - R)$. $\bar{k}_1$ is the mean number of cultural offspring per individual in the transmitting sub-population. Given that there are N learners and R role models, we get that $\bar{k}_1 = \frac{N}{R}$, and the mean for the other sub-population is 0, since they cannot pass on their trait ($\bar{k}_2 = 0$). The variance for the non-transmitters (σ_2^2) is the same, 0. The variance for the transmitting population is obtained in the same way as the variance for the full Wright-Fisher population and is $\frac{N}{R}\left(1 - \frac{1}{R}\right)$.

Inserting these values into equation D7 results in

(D8) $$\sigma_c^2 = \frac{R\frac{N}{R}\left(1 - \frac{1}{R}\right) + (N - R)(0) + \frac{R(N-R)}{N}(\frac{N}{R} - 0)^2}{N},$$

which simplifies to

(D9) $$\sigma_c^2 = \frac{N - 1}{R},$$

the solution for σ_{OTM}^2 shown in the main text (equation 6).

REFERENCES

1. O'Neill, B. Some Useful Moment Results in Sampling Problems. American Statistician, 68 (4):282–296, 2014.
