# Supplementary figures and images for: Effective population size for culturally evolving traits
Source: PLoS Comput Biol. 2022 Apr 8;18(4):e1009430. doi: 10.1371/journal.pcbi.1009430 (PMC9020689; doi:10.1371/journal.pcbi.1009430)

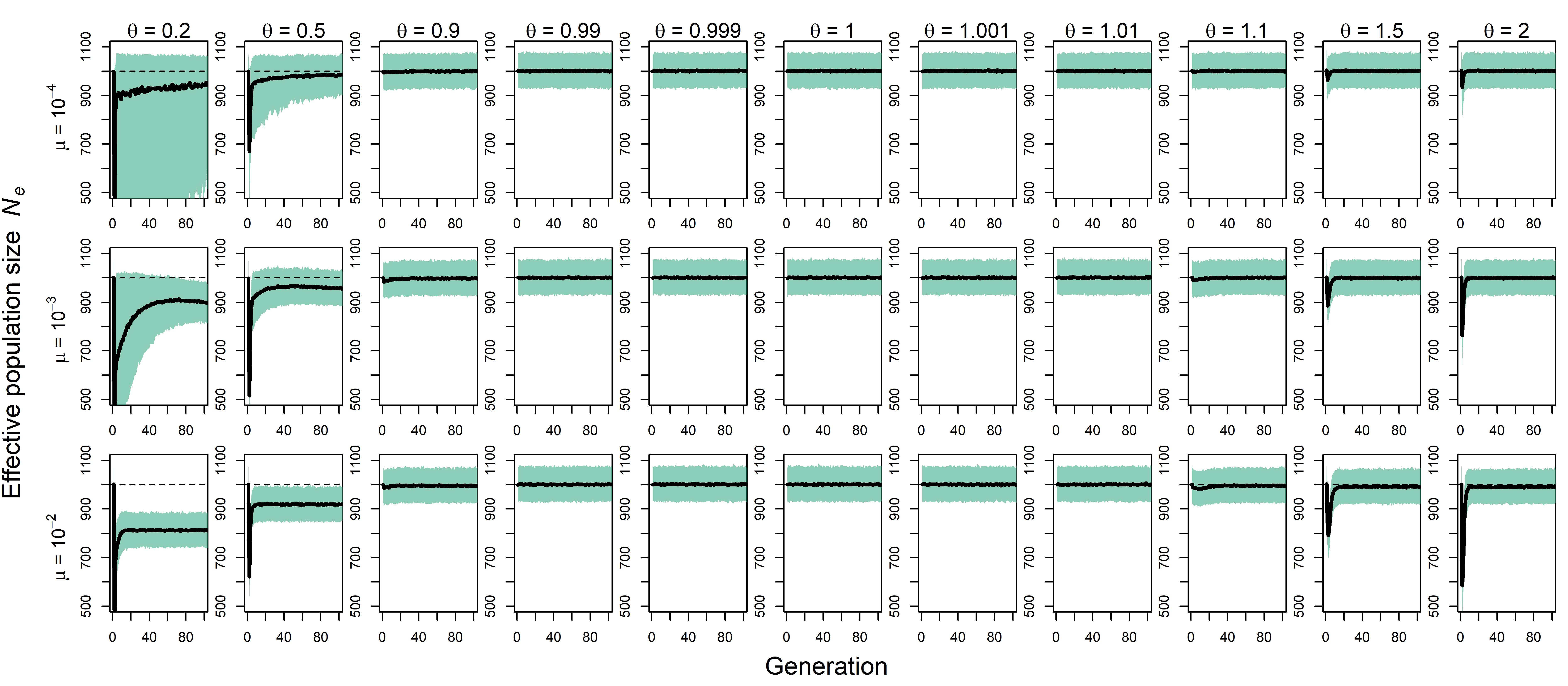

Supplement: S1 Fig — Effective population size (including 90% PIs) for different levels of anti-conformist (θ < 1), unbiased (θ = 1) and conformist transmission (θ > 1) and different innovation rates μ. Plots show trajectories for 100 generations after switch in transmission mode (1000 independent simulations; N = 1000). (TIF) [file pcbi.1009430.s006.tif]

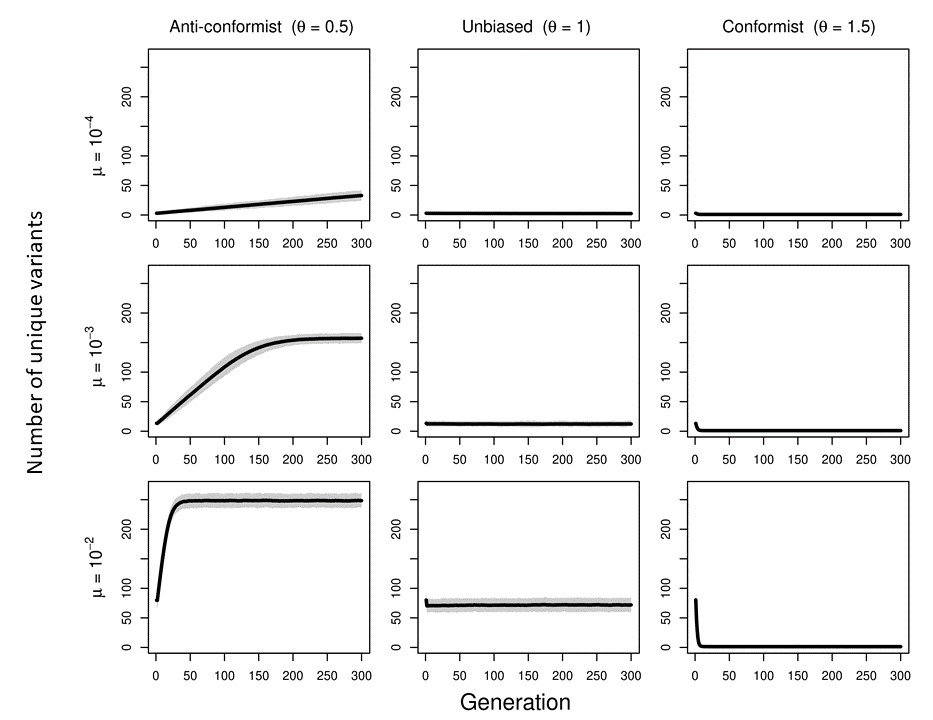

Supplement: S2 Fig — Number of unique variants (including 90% PIs) for anti-conformist (θ = 0.5; left), unbiased (θ = 1; center) and conformist transmission (θ = 1.5; right) and different innovation rates μ. Plots show trajectories for 300 generations after switch in transmission mode (1000 independent simulations; N = 1000). (TIF) [file pcbi.1009430.s007.tif]

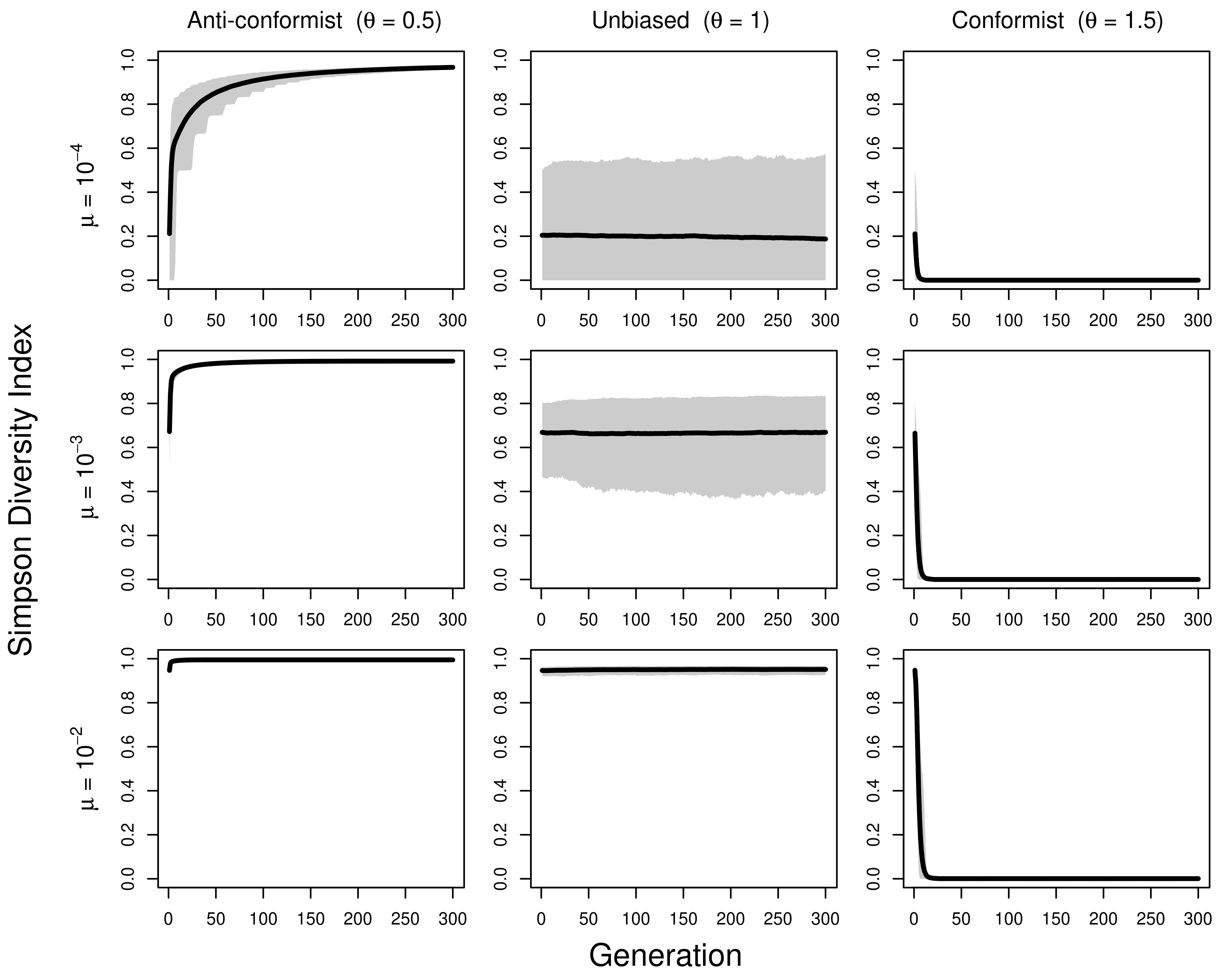

Supplement: S3 Fig — Simpson Diversity Index (including 90% PIs) for anti-conformist (θ = 0.5; left), unbiased (θ = 1; center) and conformist transmission (θ = 1.5; right) and different innovation rates μ. Plots show trajectories for 300 generations after switch in transmission mode (1000 independent simulations; N = 1000). (TIF) [file pcbi.1009430.s008.tif]

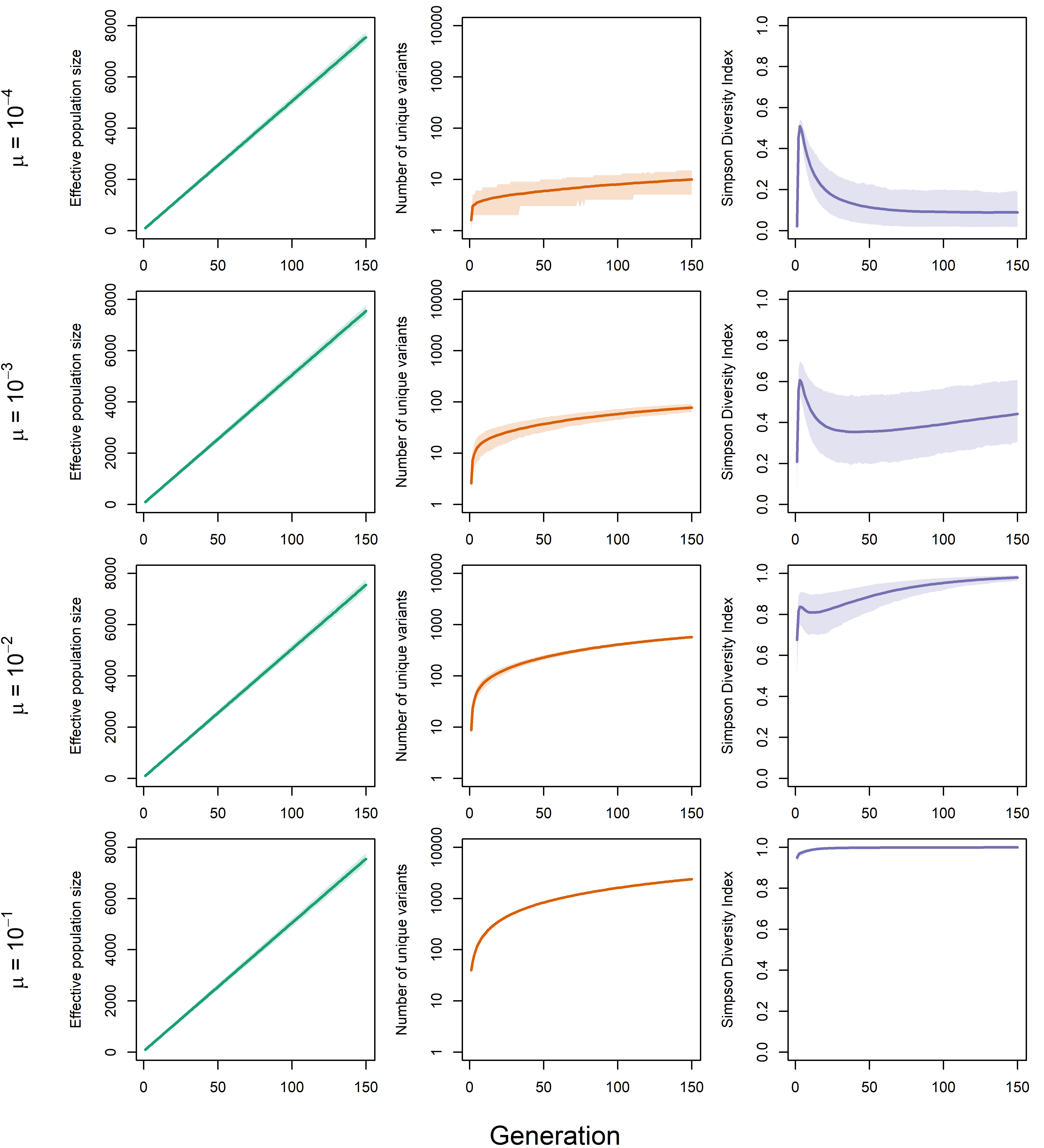

Supplement: S4 Fig — We start by letting a large source population with N = 10000 and a small focal population with N = 100 evolve separately until they reach equilibrium; each generation, we then let a fixed number of individuals migrate from the source population to the focal population and record effective numbers and diversity indices in the focal population. Effective population size (left), number of unique cultural variants (on log scale; center) and Simpson Diversity (right) for different innovation rates μ. Plots show trajectories for 150 generations after immigration starts (1000 independent simulations with 50 immigrants per generation). (TIF) [file pcbi.1009430.s009.tif]
