# Supplementary material for: Effective population size for culturally evolving traits
Source: PLoS Comput Biol. 2022 Apr 8;18(4):e1009430. doi: 10.1371/journal.pcbi.1009430 (PMC9020689; doi:10.1371/journal.pcbi.1009430)
Supplement: Attachment — Submitted filename: Response to reviewers.docx [file pcbi.1009430.s011.docx]

Dear Dr. Deffner,

Thank you very much for submitting your manuscript "Effective population size for culturally evolving traits" for consideration at PLOS Computational Biology. As with all papers reviewed by the journal, your manuscript was reviewed by members of the editorial board and by several independent reviewers. The reviewers appreciated the attention to an important topic. Based on the reviews, we are likely to accept this manuscript for publication, providing that you modify the manuscript according to the review recommendations.

As you see, Reviewer #1 recommends Accept, while Reviewer #2 recommends Minor Revision. Reviewer #2 has suggested many comments/suggestions/questions and I believe they can help improving the manuscript. Some comments are straightforward, but the others are more conceptual. Thus, I would like to invite re-submission of the revised ms and give it another round of review, at least by Reviewer #2.

Dear Prof. Wakano,

We would like to thank you and the reviewers for the careful and thorough evaluation of our manuscript. We believe that in the revised version of the manuscript we have addressed all reviewer comments and suggestions and hope that this revision further clarifies our approach and highlights the contributions of our manuscript. To aid orientation, reviewer comments are in black, our responses are in blue and new text added to the manuscript is in green.

Sincerely,

Dominik Deffner, Anne Kandler and Laurel Fogarty

Reviewer #1: This is a very clearly written paper that makes a significant contribution to the ongoing debate about the extent to which population size influences cultural diversity. The authors show that a range of different factors affect the relationship and impact the relationship between census population size and culturally-effective size, which can be both smaller and larger than the census size. To characterise the relevant factors in empirical cases will be difficult but this paper takes the theory forward significantly.

Thank you for the kind words and positive feedback on our work. We agree that the application of the concept of effective size to cultural contexts will be particularly challenging.

Reviewer #2: I’ve had a chance to read the manuscript by Deffner et al. twice and give it some thought. The revisions I suggest below are meant mainly to help clarify certain concepts or aspects of the model for the reader. In addition, I think the authors might strengthen the implications of their results—and therefore the impact of their study—by going a bit more deeply into similar critiques that have been made recently. Their study adds something new and quite interesting to the discussion. But perhaps their conclusions will have greater impact if they can communicate to the reader that they are adding to a growing body of research findings that make similar warnings regarding prior empirical tests of the so-called demographic hypothesis.

More specifically, because those tests did not consider the effective population sizes (Ne) of the traits they studied, they can't really be used to test the effect of demography on cultural diversity (or “complexity”—however that is defined). As some others have shown, their paper shows that there will be many cases in which one should not expect to see a tight relationship between census size and cultural diversity, and that this lack of a relationship should not serve as evidence against the demographic hypothesis—contrary to what previous authors have claimed. In addition, their simulation experiments show quite clearly that there are cases in which one would expect to see different levels of cultural diversity even when holding census size constant. The basic point here is that cultural diversity is a function of Ne not N, and to accurately infer Ne of any trait one needs a lot of information regarding the way in which it is transmitted. For most empirical cases—including all of those in previous “tests” of the demographic hypothesis—those data are not available. The important implication of the lack of those data is that those “tests” don’t really serve as strong tests of the demographic hypothesis at all. Furthermore, an implication for future empirical work is that we can’t really design stronger tests unless we have information regarding the way in which a trait has been passed within a population over time. My personal take here is that for almost all archaeological cases—especially those pertaining to the Paleolithic—we will simply never have the data required to properly test the demographic hypothesis because the information regarding how each trait was passed is unrecoverable. The authors could also make it clear that each cultural trait will have its own effective population number—that Ne is a property of a trait and not a property of a population through time. This is a point made by geneticists (e.g., Templeton) as well as cultural evolutionists.

Below I provide my line-by-line comments/suggestions/questions. I hope these will be useful to the authors as they refine their paper to clarify some points and to increase the impact of their results. Due to the short review period of this journal, I will have to keep my comments relatively brief and unadorned. Please note the tone is not meant to be curt or brusque; the directness of the comments is just a function of being short on time. I am impressed by this study and I think it illustrates some very interesting points that should be appreciated more widely.

Thank you for the detailed and thoughtful comments on our manuscript. We missed your relevant recently published work in our initial literature search, which we performed in 2020, and we thank you for bringing it to our attention.

Below, we respond to the comments and detail how we modified our manuscript to address them. Summarizing, we now (1) better embed our present work within the broader literature, (2) tidied up our use of terms (especially “traits” and “variants”) to make it consistent throughout the manuscript, and (3) consistently phrase the article in terms of “ cultural diversity” and more critically discuss the concept of “cultural complexity” as it is currently used in the cultural evolution literature. Thanks again for putting in the time and effort to comment on our paper in such great detail. This resulted in a substantially clearer and improved manuscript.

Comments/suggestions

General comments about the abstract:

The authors use the phrase “one-to-many transmission” throughout the paper to describe the

case in which only a subset of the members of the parental generation are available to serve as

teachers. I get the point, and that term has been used in previous cultural transmission research, but I think a better term for what they are trying to highlight here might be “some-to-many” or “subset-to-many.” They don’t model any cases in which it is just one individual teaching all of the naïve generation (R=1), which would be the literal meaning of “one-to-many” in this context. More importantly, even when the entire parental generation is eligible to serve as teachers (R=N), some of them will teach multiple individuals given unbiased cultural transmission. That is, even when R=N there is still “one-to-many” transmission via unbiased

cultural transmission in the sense that some of the members of the parental generation teach more than one naïve individual while others teach none. For these reasons, it seems to me that

something like “some-to-many” would be the better way to describe the situation where R<N.

Thank you for pointing out this potential source of confusion. We decided to stick with “one-to-many” because this is term is often used in the literature. However, when introducing this transmission mode, we now add the suggested alternative descriptions to highlight the different ways the underlying math might be verbalized.

“This transmission process, which might also be called ``some-to-many'' or ``subset-to-many'' transmission, may drastically change the variance in cultural influence and, thus, the effective population size depending on the number of role models, R.” (lines 309-311)

“For one-to-many transmission and different network structures, effective size and cultural

diversity are closely associated.” I find this to be too vague. It can probably be removed from the abstract.

Thanks! We rephrased this sentence and hope it is less vague now:

“For one-to-many transmission and different network structures, larger effective sizes are closely associated with higher cultural diversity.”

“Our results highlight the importance of carefully defining effective population size for cultural

systems and show that inferring Ne requires detailed knowledge about underlying cultural and

demographic processes.” Yes, I agree that this is what their work shows and that this is indeed the main point to be drawn from the study. They might wish to add that this adds to a growing number of recent studies that come to that same conclusion.

Thanks, we have now changed this sentence to:

“Extending previous work, our results highlight the importance of carefully defining effective population size for cultural systems and show that inferring Ne requires detailed knowledge about underlying cultural and demographic processes.”

line 26: “produce a particular cultural trait with many possible variants.” OK, so this paper is going to be dealing with a single discrete trait that has many possible variants. Are we to assume that the trait can take any one of infinitely many variants or is there a finite limit to the number of variants? Perhaps this could be made explicit.

Thanks, we now say “produce a particular cultural trait with infinitely many possible variants.”

line 27: “We now want to know whether population size affects the number of different traits

in a population.” This already veers off the path set by the previous sentence. The model has only one trait, so you want to know how pop size affects the number of variants (e.g., variant richness) expressed at that trait, not the number of traits in the population. Be consistent with the terms.

Changed to “variants” instead of “traits”.

line 28: change to “larger census size”

Done

lines 29-31: “Using a theoretical model of a cultural evolutionary process [e.g. Shennan, 2001,

Henrich, 2004, Powell et al., 2009, Fogarty et al., 2017], we conclude that larger populations

should have larger or more complex cultural repertoires.” This is muddying the waters and needs to be clarified. The models in the papers cited there are all a bit different, so I wouldn’t call them “a theoretical model of a cultural evolutionary process”. The part that is more problematic is “larger or more complex cultural repertoires”—what does that mean exactly and how does it relate to what you are interested in with this model, which is the number of variants expressed at a single trait? Does a larger cultural repertoire mean more traits or more variants per trait? Be specific. And what exactly makes a cultural repertoire more “complex” in this context? Henrich models skill level—how does that value directly relate to the complexity of a cultural repertoire? I would say it is unclear what it actually means in terms of “complexity.” Shennan’s model shows an increase in mean fitness under some conditions—does that mean a variant with higher fitness must be more “complex?” Probably not—so long as there are examples of “simple” solutions that get the job done better than more complex solutions. I don’t think Shennan ever made that association with complexity. As best I can tell, this supposed association between the values Shennan was modeling and notions of “complexity” come mainly from Collard et al.’s (2005) assertion that the two can be treated as one and the same. I think that assertion is ill-advised and should not be propagated further. Or at the very least, if “complexity” is going to be used it must be defined and the relationship must be made explicit. Otherwise, people just keep using the term as if it has a well-defined meaning when in fact it does not in this context.

Suggested rewrite: “Under conditions in which a selectively neutral trait with infinitely many

variants is passed via unbiased cultural transmission, one ought to expect population A to

display a greater number of variants than population B at that trait.”

Thanks for this suggestion. We agree that the previously cited models are diverse in terms of their assumptions and underlying mechanisms, which might lead to confusion. We also agree that our model does not directly address cultural repertoire size or the (ill-defined) notion of cultural complexity, so we changed this sentence as follows:

“Based on some theoretical model of a cultural evolutionary process, we might be led to expect population A to display a greater number of variants than population B at that trait.” (lines 29-31)

lines 32-33: “Can we expect to find this demographic relationship in data on census population

sizes and cultural repertoire sizes from both populations [e.g. Oswalt, 1976]?”

I find this confusing, partly because it switches from number of variants at a single trait to cultural repertoire size, as if they are the same thing. Perhaps it can be rewritten with greater focus as: “But will the number of variants expressed by each of our hypothetical populations A and B differ due to their different census population sizes?” I don’t think there is a need to cite Oswalt in this case because his units are different than what we call a cultural variant. I think this sentence could also just be deleted if the sentence above it is changed.

I would recommend to delete: “The answer is that—regardless of how good the model is—the

relationship is unlikely to be found unless these real populations are identical in some

evolutionarily important ways.”

lines 35-38: Suggested rewrite: “However, two populations with the same census size might not display the same number of cultural variants at a trait if they are marked by different

demographic histories or if the way in which the trait is transmitted differs among the

populations. In other words, the number of variants displayed in the two populations of

equivalent census size will differ if the effective population size of the trait in question differs

between the two populations. Many factors—not just census size—play a role in defining the

effective population size of a cultural trait (e.g., Premo 2016, 2021).”

Thanks for these suggestions. We have now re-written this section:

“Two populations with the same census size might not display the same number of cultural variants at a trait if they are marked by different demographic histories or if the way in which the trait is transmitted differs among the populations (see [Premo, 2016] and [Premo, 2021]). In other words, the number of variants displayed in the two populations of equivalent census size will differ if the effective population size of the trait in question differs between the two populations. In such cases, the populations are not directly comparable, except through their relation to a simpler model—through their effective population size.” (lines 34-41)

line 47: Change “how many cultural traits we expect” to “how many variants we expect”. You

are interested in the number of variants at a single trait not the number of traits in a population. Be consistent with the terms.

Done

line 49: Change “expected number of traits is” to “expected number of variants is”.

Done

line 50: Change “see on average 41 traits in a given generation” to “see on average 41 variants

in a given generation”.

Done

line 53: “Using census numbers or more informal definitions of effective size will produce incorrect results.” I get your point but this could be sharpened to: “Employing census size—

rather than effective population size—to predict number of variants per trait is bound to lead

one astray in any empirical population that violates one or more of the conditions of the Wright-Fisher model.”

We have now re-written this section as follows:

“Employing census size or more informal definitions ---rather than effective population size---to predict cultural diversity is bound to lead one astray in any empirical population that violates one or more of the conditions of the Wright-Fisher model.” (lines 56-58)

line 57: “…the effects of drift, innovation, migration (intergroup transmission), and selection

in…”

Changed

line 62: “…are possible. Ne can also exceed census size N (Crow and Kimura 1970)—a…”

Changed

lines 66-69: 100% agreed!

Thanks!

lines 86-88: “In a first formal treatment for cultural evolution, Premo [2016] used simulation models to investigate effective population size and its relationship with cultural complexity.”

Although complexity is in the title, I don’t really use that term when describing my own results. I’d rewrite that as “to investigate the relationship between census size, effective population size, and the rate of change in mean skill level under non-ideal conditions.”

Thanks, we have now re-written this sentence accordingly.

lines 88-90: Following on to the comment above, edit this to read: “In the context of models by Shennan [2001] and Henrich [2004], the results show that natural and cultural selection can

weaken the relationship between census population size and the rate of change in mean skill level.”

Changed

“did not formally derive an appropriate measure for cultural effective size”. It is true that I did not come up with any new derivation on my own in that paper, but I did employ the inbreeding effective number, which is appropriate for a cultural trait if you have data on Vk and k_bar, both of which I collected from simulations.

Line 92: In a paper that was published earlier this year in Journal of Archaeological Method and Theory, I investigated the relationship between Ne and the coefficient of variation of a continuous trait passed via different combinations of vertical, prestige, unbiased, and conformist biased transmission in a finite population of constant size. That part of the paper, which has a bit of overlap with this manuscript even though a major difference is that I dealt

with a continuous trait and they deal with a discrete trait, could be briefly reviewed here as well. For example, I (Premo 2021:523) make some of the same points that these authors make

later in their paper: 1) a trait’s effective pop size can be infinitely large under vertical transmission, 2) under conformist biased transmission where everyone learns from just one teacher (this would be the most extreme form of one-to-many transmission), the effective pop

size of the trait is approximately 1, and 3) under unbiased cultural transmission, the effective

pop size of a cultural trait in a finite population is very close to N (this same point is made in

Premo 2016:608). Another point made in both Premo (2016) and (2021) that might be worth

mentioning in this section of the paper is that every cultural trait has its own effective number

depending on how it is transmitted. This adds a further complication, because each trait needs

to be studied on its own and data regarding how trait X is passed may not be pertinent to trait Y even if both traits are in the same population.

Thank you for pointing us to this work that we missed during our initial literature search. We have now included the following section:

“Further, Premo (2021) studied the accumulated copying error model (ACE) and investigated the relationship between the effective size and the coefficient of variation of a continuous trait passed via different transmission pathways. Importantly, both Premo (2016) and Premo (2021) emphasized that every cultural trait has its own effective number depending on how it is transmitted. This adds a further complication, because each trait needs to be studied on its own and data regarding how trait X is passed may not be pertinent to trait Y even if both traits are in the same population.” (lines 97-103)

line 96 “different modeling approaches to investigate” I’m not sure what “different modeling approaches” means in this case. It seems the general modeling approach is the same (simulation) but the assumptions included in the model vary. Perhaps this could be rephrased to avoid causing confusion.

We use “different modeling approaches” in the sense that we provide analytical solutions (for OTM transmission) alongside simulation results for other situations. To avoid confusion, we now changed this to “mathematical models and simulation”.

line 97: “namely one-to-many” Again, here I might go with “some-to-many” or even “subset-to-many” instead of one-to-many, but I leave that up to your discretion.

We now include all of these descriptions where we introduce OTM transmission. See answer above.

line 100: “we relate effective numbers to the emerging levels of cultural diversity.” Yes, so this keeps the focus on diversity—i.e., number of cultural variants—of a single trait rather than on the vague and problematic notion of repertoire “complexity” or “size”. I think this is exactly the right focus.

Thanks, we’re glad you agree with our general focus on diversity and our terminology has been tightened throughout.

line 126: maybe edit to: “and the allele either becomes fixed or goes extinct with equal probability given a 0.5 initial frequency (Wright 1931)”

Changed

line 129: “…it takes for isolated populations to diverge.”

Changed

line 131: I’d suggest deleting “and the allele will go to fixation/extinction.”

Done

line 138: rewrite to emphasize that Ne is a property of a locus within a given population, not just a population—that is, different loci can have different effective population numbers within

the same population: “…genetic variants maintained at a locus for a given population.”

Changed

Equation 1: Yes, this checks out. And this is the same equation used in Premo (2016, eq 1) and

(2021, eq 8). Because I hold population size constant in those papers, there isn’t a difference between inbreeding and variance effective pop size. But in this paper, they will need both equations if they are going to vary population size though time.

Exactly. We need to differentiate between the formulations because we discuss cases where population sizes are not constant over time.

Equation 2: The authors are correct to point out that the variance effective number is affected

by the size of the offspring generation more than the size of the parental generation. They might be able to better illustrate this point by providing the equation that uses Nt rather than Nt-1. The Crow and Kimura (1970) book they cite in this section of the paper presents such an equation in Table 7.6.4.1 on page 362. Although it introduces a new term, alpha, perhaps using

that equation (Eq. 7.6.3.26 from Crow and Kimura 1970) would make the distinction between

the two measures even clearer. Just a thought.

Thanks for this idea. We are hesitant to include that equation without mentioning or using alpha_{t-1} anywhere else, as this might only lead to more confusion. We have now improved the explanation (see answer below) and point to the alternative equation in the Crow and Kimura (1970) book. We hope that this is sufficient but we are happy to revisit the issue in the next revision in case the reviewer or editor feels strongly about this issue.

lines 160-165: I find this section a bit confusing, so I can imagine it will be very difficult for a

reader who hasn’t read the primary literature on this difference. It should probably be rewritten or left out.

Thanks. We have now tried to express the contrast between variance and inbreeding effective numbers more clearly:

“To understand why, imagine two parents, one carrying allele A, one carrying allele B, randomly producing a large number of offspring. In this scenario, two offspring will have a large probability of sharing the same parent (resulting in relatively low inbreeding effective size), but the frequency of both alleles will still be close to 50% in the offspring generation (resulting in relatively high variance effective size). If, on the other hand, a large number of parents produces only a small number of offspring, there will only be a small probability that two offspring share the same parent, while allele frequencies will differ greatly among generations (i.e. high inbreeding effective size and low variance effective size).” (lines 171-179)

Equation 3: Yes, again, this equation checks out. This appears as Equation 9 in Premo (2021).

Great!

lines 190-193: “Thus, it is clear that any process that systematically alters the way in which cultural role models are chosen, through the mode of transmission, demography or social network structure, will change the variance in cultural influence and the effective population

size for culturally evolving traits.”

Yes, indeed, this is a fundamental but very important point! Perhaps this would be the best place to add a sentence or two to point out that if different traits are passed via different mechanisms within the same population, they will each exhibit difference effective population

sizes even though the population is marked by the same census size.

Thanks for the great suggestion: We have now included the following sentence:

“As noted before, if different traits are passed via different mechanisms within the same population, they might each exhibit different effective population sizes even though the population has the same census size.” (lines 206-208)

line 206: “until it reaches its equilibrium state” How is that eq state defined here—expected number of variants?

Thanks for this opportunity to clarify. We have now included an additional section in the supplementary material where we detail the burn-in procedure and point to this in the main text (line 222):

“To remove any potential effect of initial conditions, before we start recording values, we first let the system evolve through unbiased transmission until it reaches its equilibrium state. To achieve this, we set up two separate populations, population A with maximum diversity (i.e., each individual carrying a different variant) and population B with 0 diversity (i.e., each individual carrying the same variant). We then recorded the Simpson Diversity Index in each generation (see section on simulation set-up in the main text) and let the system evolve until diversity in population B first exceeds diversity in population A. This procedure ensures that the system has reached its equilibrium diversity value which is also confirmed by the observation that all simulation results reported in the main text were identical for both original populations.”

line 216: suggested edit: “where high scores indicate high cultural diversity (a large number of

variants each displayed at a low frequency) and low scores indicate low cultural diversity (few

variants, some of which are displayed at relatively high frequencies)”

Done

line 283: edit: “In addition to unbiased transmission, we consider two other transmission processes: some-to-many transmission and frequency-dependent transmission.”

Done

line 288: Again, I’d suggest considering whether to call this something like some-to-many or

subset-to-many rather than one-to-many.

See answer above.

line 292: edit: “…R individuals are eligible to serve as models for the naïve generation and pass on their variant via unbiased transmission and N – R individuals are not eligible to pass on their variant at all.”

Changed

“we have a transmitting sub-population of size R” I’m not sure whether this detail matters, but note that the number of individuals that actually transmit their variant (the transmitting sub-population) will be less than R because R is the subset of teachers that could be chosen at random via unbiased transmission not the number that actually are chosen as teachers. Only about 63% of the eligible set will be chosen, which means that the actual number of individuals who transmit a variant is lower than the sub-set R.

Thanks. We think this nuanced point is much clearer now that we implemented the previous edit you suggested (original line 292).

Equation 7. I like that they were able to derive this equation, but it might be worth mentioning

that it won’t be exact for small finite populations. For N=10 and R=2, I could get something like this under unbiased transmission: (7,3,0,0,0,0,0,0,0,0) which has a variance of 5.33 and would yield an Ne of 9/5.33=1.69 rather than 2. For N=10 and R=3, I could get something like this under unbiased transmission: (7,2,1,0,0,0,0,0,0,0) which has a variance of 4.89 and would yield an Ne of 9/4.89=1.84 rather than 3. In short, it looks like equation 7 would be likely to overestimate Ne in finite populations. I think the equation is still useful, but perhaps this caveat could be noted somewhere.

Thanks for this comment. It draws attention to some extra technical information that would be helpful here. The equations calculate expected results and are unlikely to accurately describe the effective size in any single generation in any single simulation run or population snapshot. In the example of N=10, and R=2, we have 9 possible combinations of parents (so individual R1 could have 9 offspring and individual R2 could have 1; individual R1 could have 8 offspring and individual R2 then has 2, etc… ), each of these has a certain probability of occurrence in any particular generation which can be calculated using the fact that there are ten offspring, each of whom has an equal probability of being born to either parent R1 or R2. The sum of the product of these probabilities and the relevant exact values of the variance yields the variance calculated by the equation. We note also that we have calculated population variances, not sample variances where Bessel’s correction is applied. This matters less and less for higher N but for small N e.g. in the example of (7,3,0,0,0,0,0,0,0,0) it plays a role. Further, we note that the reviewer's point applies equally to the more established case of equation 3 and our relatively simple extension in equation 7. Below is some R code to illustrate the calculation.

library(pracma)

#Example:

N=10

R=2

\# Individual generation possible realisations: parents = (number of offspring of parent 1, number of offspring of parent 2)

Combs <- list()

for (i in 9:1) Combs[[i]] <- c(N-i,i)

\# Probability of these outcomes can be calculated as e.g. for the first option nchoosek(10,9)/(2^10). Similarly we

\# can get a vector of these probabilities:

probvec <- c()

for (i in 9:1) probvec[i] <- nchoosek(10,i)/(2^10) #probvec = c(0.0098,0.0439,0.1172,0.2051,0.2461,0.2051,0.1172,0.0439,0.0098)

\# Each of these outcomes has an associated specific population level offspring variance, e.g. var(9,1,0,0,0,0,0,0,0,0)*((N - 1) / N)

varvec <- c()

for (i in 1:9) varvec[i] <- var(c(Combs[[i]], rep(0,8)))*((N - 1) / N) #varvec =c(7.2,5.8,4.8,4.2,4,4.2,4.8,5.8,7.2)

\# The sum of the product of these is then given by

X = sum(probvec*varvec)

#This is the expected offspring variance, which is used in equation 7,cN-1/X

NE_1 = (N-1)/X	#Which is very close to R=2.

line 314: “(census size is held constant at 1000).”

Changed

Figure 2: Very clear figure; nicely done! As an aside, this shows me the number of unique

variants is probably the more useful measure of diversity.

Thanks very much!

line 330: What does “partly substantial” mean? Rewrite that part with a better term.

We have now changed this to “under certain circumstances substantial”

line 331: “the severity of the peak” But aren’t these valleys rather than peaks, or am I looking at the wrong thing in Fig. 3?

Thanks for pointing this out. We have replaced “peak” by “trough”.

Figure 3. Another very interesting and clear figure! It would be interesting to see the cultural diversity data for these simulations in another figure. I’m interested in whether you see a *greater* number of variants in the lower left corner even though Ne is *lower* under anti-conformity? Clearly, the number of variants would be much lower than expected for an Ne of 1000 under conformity (right hand column), but it might be worth showing that in a figure that

plots number of cultural variants rather than Ne on the Y-axis.

Thank you for the suggestion. We have now included the proposed figures in the supplementary material (Figs. S2, S3) and refer to them in the main text. Your intuition was exactly right, there is a greater diversity in the lower left with anti-conformity and high innovation rates.

“In appendix 6, we also plot time trajectories for the Simpson diversity index (Fig. S2) and the number of unique variants (Fig. S3). As expected, conformist (anti-conformist) transmission decreases (increases) Simpson diversity and number of variants over time, irrespective of the observed time dynamic of the effective number.” (lines 378-382)

line 346: “But in contrast to the conformist situation, the effective population size at equilibrium is greatly influenced by the innovation rates; per definition, innovations are rare

and, thereby, the target of anti- conformity. The higher the innovation rate, the more likely a

variant is present the population at low frequency and, therefore, the higher the differences

between individuals in their cultural influence.” That is a cool result! I don’t think I could have predicted that one, but it makes sense to me now.

Thanks!

line 388: “In contrast, for the variance effective size, only the number of learners matters such

that changing the number of role models has no effect.” Really, does it have no effect at all? Is that statement generally true or just true of the way you have modeling exchange here? It seems that decreasing the number of potential role models from N to 1 would surely have a large effect even if the naïve generation is much larger or smaller than the paternal generation, no?

Thank you, this was much too strongly phrased on our part. We have softened the language. It now reads:

“In contrast, the variance effective size is more directly related to the number of learners than to the number of role models.“ (lines 414-416)

We now also specify the relevant sections of Crow and Kimura (1970, pp. 345-359), which we rely on in our explanation.

line 393: edit suggestion: “Increasing cultural exchange causes the greatest increase in Simpson

diversity and the number of unique variants when exchange rates are low.”

Changed

line 399: “So far we have considered panmictic (or “well-mixed”) populations” I don’t think the case where R<N would be considered panmictic because N-R individuals are not allowed to pass on their trait.

Thanks. We have now deleted this sentence.

line 406: edit suggestion: “Fig. 5, second row, shows the way in which transmissions are

structured affects the trait’s effective population size.”

Changed

line 409-413: “As every individual has the same probability p of being connected to any other

individual, the number of links are binomially distributed and there is no systematic difference

in individuals’ probability to pass on their cultural variants. Density also does not affect levels of cultural diversity such that strongly interconnected populations are not more diverse if connections are random.” This is another cool finding that I had not anticipated. I figured lower density would lead to higher diversity, but apparently that is not the case in a random network. This is good to know even though there probably aren’t many truly random networks in humans.

Thanks for this comment. Yes, it is an extreme case but hopefully an informative finding.

line 418: “This increased variance in cultural influence results in substantially lower effective

numbers and also lower levels of cultural diversity.” But that isn’t true for all the values you tried here. Diversity is affected only when pi is quite high.

The scale of the y-axis hides a subtler decrease in diversity for lower values of pi because the number of variants is shown on a log scale. We now make this clearer in the figure legend. Also note that the same values of pi that lead to more abrupt shift in Ne also result in faster change in cultural diversity.

line 423: “This demonstrates that Ne can also exceed N in cultural systems.” Yes, good point. Another example of this is provided by vertical transmission (see Premo 2021:523). Under vertical transmission, Vk = 0 and the inbreeding Ne is infinitely large (Crow and Kimura 1970:361).

Thanks for this comment. As Vk approaches 0, the effective number becomes very large. For Vk=0, our formulation of Ne is undefined.

line 428: replace “goes up” with “increases”

Done

line 432: “…for cultural traits.”

Changed

line 434 “…of population structure affect the effective population size of a cultural trait. Some-to-many unbiased cultural transmission and …”

Changed

line 437: “when the system is out-of-equilibrium” What does that mean in this context? You could not have effects when it is in equilibrium, so I’m not sure what to make of this statement.

By “out-of-equilibrium”, we mean that we switched the dominant transmission mode and the cultural system has not yet reached its new equilibrium diversity level. However, as observed for anti-conformity (as well as one-to-many transmission), there can be effects of transmission modes on the effective size of a trait even if the cultural system has reached equilibrium.

line 440: “This implies that considerable precision and caution is needed in defining cultural

effective sizes.” I agree with this sentiment, but I think the combination of “precision and caution” might be a bit awkward. At the very least, this point deserves a few more sentences to fully flesh out what is meant by this warning.

Thanks. We have now changed this sentence to:

“This implies that considerable precision is needed in applying an appropriate cultural effective size formulation.” (lines 465-466)

lines 444-455: This entire paragraph reverts to talk of “cultural complexity,” which as I argued above is not a very useful concept. What is more, the results presented above deal with diversity, not complexity, so this feels like a sharp turn away from the results and towards a less concrete and less useful term. If this paragraph is to be kept it might be worth adding a critique that cultural “complexity” is not well defined and therefore problematic. The term sounds “sciency” but in the end it isn’t very useful because it is very hard to pin down. Number of variants, on the other hand, is not hard to pin down—although that doesn’t mean it will be easy to measure in archaeological material.

See answer below

line 456: “These inconsistent findings do not necessarily refute the theoretical models but

might instead reflect a poor correspondence between theory and empirical tests.” Yes, right on point. I could not agree more. In fact, that was the point of the “Revisiting empirical tests” section of Premo (2016:617-620).

Thanks for pointing out these crucial issues. We have now included an additional paragraph where we critically discuss how the poorly defined notion of cultural complexity has been employed in the theoretical and empirical literature and that we advocate for the better defined concept of cultural diversity instead. We are now also referring readers to the “Revisiting empirical tests” section of Premo (2016).

“These inconsistent findings do not necessarily refute the theoretical models but might
instead reflect a poor correspondence between theory and empirical tests [see also section
on “Revisiting empirical tests” in Premo, 2016]. First, “complexity” itself is notoriously
difficult to pin down [Mitchell, 2009] and “cultural complexity” is hence conceptualized very
differently across different models and empirical investigations making it difficult to draw
inferences. In Henrich’s model, for instance, cultural complexity is simply represented by
the value of a continuous variable *z* [Henrich, 2004], whereas other models describe cultural
complexity as cultural repertoire size [Creanza et al., 2017, Fogarty et al., 2017] or the level
of innovations in a tree-shaped cultural landscape [Derex et al., 2018]. Similarly, empirical
studies use measures as diverse as toolkit size [Kline and Boyd, 2010], number of techno-
units [Oswalt, 1976, Kline and Boyd, 2010, Collard et al., 2005] as well as paper plane
flight distances [Fay et al., 2019]. Instead of the poorly defined notion of cultural complexity, in the present work, we rely on the well-defined and workable concept of cultural diversity. Here, we have demonstrated that obtaining correct and comparable values for the population
size in complex cultural scenarios is not straightforward either. In order for the results
from theoretical models to apply correctly to empirical cases, we need to ensure that model
parameters are correctly translated into measures from complex real-world scenarios.” (line 482-498)

line 471: “In this case, larger local clusters or more links between clusters somewhat counter

intuitively reduce the effective population size even though individuals now share ties with

more potential cultural models.” This is a good point to address, as I think most people would assume that greater connectivity would yield greater diversity. But that isn’t necessarily the case. For instance, pop geneticists have shown that a structured population can exhibit a larger Ne than a panmictic pop, holding census size constant. Structure is introduced by cutting connections between individuals in non-random ways.

Thank you. We agree that this is an important point that violates many peoples’ first intuitions that greater connectivity should always lead to greater diversity.

line 486: edit: “Overall, these results highlight that census numbers cannot generally be relied

on when evaluating hypotheses about the effects of demography on culture. The diversity of a

trait is a function of its effective size not its census size, and inferring effective population sizes

requires detailed knowledge about underlying cultural and demographic processes.” Yes, this is right on the mark!

Thanks very much!

line 492: Yes, agreed, there is so much work that needs to be done before jumping to conclusions about transmission mechanisms and this paper does a good job of highlighting what some of the things are that we need to gather data on before interpreting the results of empirical tests.

Thanks again!

line 500: “should be related to cultural complexity.” I’d steer clear of complexity and just stick with diversity. The statement is still true with diversity.

Done

line 501: “In relatively egalitarian societies, in contrast, where all individuals are equally likely to transmit their ideas and behaviors, census size could be a reasonable approximation of effective size.” I don’t think that is true. Even in a relatively egalitarian society, not everyone will be seen as equally capable of making a stone spear point or procuring medicinal plants. There are still experts among egalitarian societies and those experts might skew the k values for certain traits and not others. Instead of speculating that we need not worry about the difference between N and Ne in egalitarian societies (which I think is bad advice that might be followed by archaeologists working on hunter-gatherers), I think this is another place to remind the reader that one must really focus on one trait at a time because all of them might be different—even in egalitarian societies.

Thank you for this suggestion. We have now weakened our claim and discuss the importance of studying cultural phenomena on a trait-by-trait basis.

“In societies where all individuals are more or less equally likely to transmit their ideas and behaviors, census size might be a better approximation of effective size. However, note that also most egalitarian societies are characterized by substantial degrees of domain expertise expected to skew cultural influence for certain traits. This again highlights that cultural phenomena are to be studied on a trait-by-trait basis.” (lines 539-541)

line 507: “Finally, if social network structure prevents ideas from flowing freely through the

community, census numbers might still be appropriate to test demographic hypotheses for traits in which social network connections are random.” OK, I see the point here. But isn’t it the case that density in the random network does not affect Ne precisely because the random network does not prevent ideas from flowing freely. So, I guess I’m saying there is no random network that could prevent ideas from flowing freely. To fix this I’d simply delete “if social network structure prevents ideas from flowing freely through the community,”.

Thank you, this has been changed to “Finally, if social network structure imposes limits on cultural transmission, census numbers…”

line 512: edit: “…of a cultural trait depending on…” As I understand it, there is no such thing as the effective size of a “cultural population,” Ne is a characteristic of a trait, not a population per se.

Thanks, this is changed now.

I hope at least some of these thoughts and suggestions are useful to the authors as they work

on improving what is already a very interesting and worthwhile paper.

I waive my anonymity.

Sincerely,

Luke Premo
