# Supplementary material for: Effective population size for culturally evolving traits
Source: PLoS Comput Biol. 2022 Apr 8;18(4):e1009430. doi: 10.1371/journal.pcbi.1009430 (PMC9020689; doi:10.1371/journal.pcbi.1009430)
Supplement: Attachment — Submitted filename: Response to reviewers_R1.docx [file pcbi.1009430.s012.docx]

As you will see, the reviewer (Luke Premo, he waived his anonimity) found a big improvement in the revised manuscript. In addition to many editorial changes, he suggests some points to improve the manuscript even better. I will send you back the manuscript so that you can make changes. Ater receiving your final version, I will be glad to accept the paper.

Dear Prof. Wakano,

Thank you again for the thorough evaluation and fast processing of our manuscript. We have now addressed all additional comments and look forward to seeing this published in PLOS Computational Biology. Again, reviewer comments are in black and our responses are in blue.

Sincerely,

Dominik Deffner, Anne Kandler and Laurel Fogarty

Reviewer #2: The revised paper is a big improvement on what was already a very interesting study. The authors did a nice job addressing the concerns I had shared in my initial review. I have included one more round of suggestions and comments that the authors might like to address in their final version. Most of the suggestions are merely editorial, but a few are substantive. My kudos to the authors! I hope some anthropologists find this article.

Thank you again for your time and effort which greatly improved this manuscript.

Line by line suggestions for the final version; these are most cosmetic/grammatical to help clarify the writing:

line 34: “…unless the ways in which the trait of interest is passed in these real populations are otherwise identical in evolutionarily important ways. Put differently, two populations with the same census size…”

Changed.

line 52: “…the expected number of unique variants of a cultural trait passed via ______ transmission with a copy error rate of ______ is 223.”

Changed. For this example, transmission was unbiased and the rate of copying error was 0.1.

line 71: The human population is now close to 8 billion (shockingly), not 7 billion. The 2007 publication cited to support the 7 billion number is now 15 years old and not very close to “the time of publication.” I’d recommend updating this number and dropping the 2007 citation.

Thanks for pointing this out! We’ve now updated the number; the 2007 citation is for the study investigating genetic N_e in humans, not for the current population size.

line 200: “…we now consider cultural transmission rather than biological inheritance and…”
Changed.

line 201: “…individuals to which role models transmit their cultural variant.”

Changed.

line 298: “…variants can be passed between individuals,…”
Changed.

line 305: “…transmission, under frequency-dependent transmission this number emerges from the interplay between the transmission mechanism and the frequency spectrum of the cultural variants.”

Great suggestion, thanks.

Figure 2 is a great figure. That should be in a textbook.

Thanks very much, maybe you should write one!!

line 381: “…trajectories for the number of unique variants (Fig. S2) and the Simpson diversity index (Fig. S3).”

Wrong order, indeed. Thanks for correcting this.

line 384: Cool new result. I think this is a great illustration of how cultural transmission can create a big disconnect between Ne and cultural diversity in ways that don’t really happen in genes, and this fits with the authors’ larger goal in this paper. Adding one more sentence here tying figures S1 and S2 together would make an even greater impression and really drive home this point. If it were me, I would heavily emphasize this finding. I would recommend adding something like this to make this important point clear: “In fact, our results show that anti-conformist biased transmission yields *greater* cultural diversity than unbiased transmission (Figs. S2 and S3) even though a trait passed via anti-conformist biased transmission has a *lower* effective number than one passed via unbiased transmission (Fig. S1), holding all else constant.”
Boom!
Thank you very much for this great suggestion. We agree that this is definitely a finding that deserves greater emphasis.

line 439: “When network connections are random, density does not affect cultural diversity.”
Changed.

line 444: “…a greater number of individuals…”
Changed.

line 460: “…populations (or traits) where it would otherwise…”
Changed.

line 469: “…leave a trait’s Ne unchanged, scale-free…”
Added.

line 471: “…several patterns of cultural change,”

Thanks for spotting this typo!

line 489-490: Well yes, z is a continuous variable, but Henrich clearly goes a bit further than that. Henrich is clear that z represents a skill that is difficult to copy even when learning from the previous generation’s best practitioner. This idea that z represents a difficult to learn skill motivates his use of the Gumbel distribution. So, in Henrich’s model z is a bit more than “just” a continuous variable, which could be represented by a normal distribution or a uniform distribution or some other distribution. The notion of skill is central to what z means in his model and how noise in transmission is represented with the Gumbel distribution—calling z merely a “continuous variable” misses this point. Others working around the same time seem to have taken the notion that greater skill is the same thing as greater complexity (I don’t think that is necessarily true, and the proposed connection between skill and the size of one’s toolkit was always a mystery to me—toolkit size is a function of many things, "skill" probably among the least important) and it rather quickly spun out of control with a series of empirical "tests" that made use of others’ data and others’ units of analysis that were no longer really addressing anything of substance, at least regarding the effects of “demography” on “complexity”. It is no wonder many of these studies yield confusing and contradictory results—looking back, it seems to me they were interpreting noise rather than signal. In sum, I think the authors can describe z better than just a continuous variable—it was a very particular kind of continuous variable—based on the notion of a transmittable skill—and that helps explain some of the work that followed (and in my opinion, why it went down the wrong track).

Thank you again for your thoughts! We agree that the concept of z in Henrich (2004) goes beyond just any arbitrary continuous trait. To avoid causing further confusion in the literature, we have now rephrased the description to: “In Henrich's model, for instance, cultural complexity reflects a difficult to learn skill and is represented by the value of a Gumbel-distributed continuous variable z….” (lines 492-493).

line 503: “…size of the population and the effective size of the cultural trait in question can diverge.”
Changed.

line 505: “…between the census of the population and the effective size of the cultural trait can either…”

Changed.

line 511: “…reduce the effective population size of the cultural trait even though—or rather, because—individuals now share ties with more potential models.”

Great addition, thanks!

line 519: “…on the effective population size of the cultural trait.”
Changed.

line 520: “…dynamics, but rather that its…”
Thanks, changed!

line 526: “It is the effective size of the trait that matters…”
Changed.

line 528: “…demographic processes on a trait-by-trait basis.”
Changed.

line 538: “…cultural influence of a given trait is highly skewed…”
Changed.

line 539: “…related to cultural diversity at that trait.”
Changed.

line 541: “…be a better approximation of the effective size of a cultural trait.”
Changed.
line 546: “…proxies for the Ne of a cultural trait as long as…”
Changed.

line 548: “…cultural systems are out of…”
Thanks for spotting this typo!

line 551: “…connections are random.”
Changed.

As usual, I waive my anonymity.
Sincerely,
Luke Premo
